# Optimal cut-off for neutrophil-to-lymphocyte ratio: Fact or Fantasy? A prospective cohort study in metastatic cancer patients

**DOI:** 10.1371/journal.pone.0195042

**Published:** 2018-04-06

**Authors:** Yann-Alexandre Vano, Stéphane Oudard, Marie-Agnès By, Pauline Têtu, Constance Thibault, Hail Aboudagga, Florian Scotté, Reza Elaidi

**Affiliations:** 1 Department of Oncology, Hôpital Européen Georges Pompidou, Paris, France; 2 University of Paris 5 Descartes, Paris, France; 3 Cancer Immune control and Escape, UMRS1138, Cordeliers Research Center, Paris, France; 4 Angiogenesis and Immunotherapy laboratory, PARCC, INSERM U970, team 10, Paris, France; 5 Department of Oncology, Centre Hospitalier Universitaire Bretonneau, Tours, France; 6 Department of Pharmacy, Hôpital Européen Georges Pompidou, Paris, France; 7 Association pour la Recherche de Thérapeutiques Innovantes en Cancérologie, Department of Oncology, Hôpital Européen Georges Pompidou, Paris, France; University of North Carolina at Chapel Hill School of Medicine, UNITED STATES

## Abstract

This study assessed the prognostic value of pre-treatment neutrophil-to-lymphocyte ratio (NLR) in patients with metastatic solid tumors. Clinical and biological data for patients with metastatic solid tumors treated in an oncology outpatient department and prospectively followed by a call center (PROCHE program) between January 2008 and December 2011 were analyzed. All patients with an NLR value within 28 days before the first cycle of first-line of chemotherapy were included (cohort 1). To assess influence of chemotherapy line on NLR prognostic value, data from patients treated with later chemotherapy lines were also analyzed (cohort 2). Adjusted multivariate Cox regressions with or without non-linear and time-dependent effects were performed. Optimal NLR cut-off was investigated by time-dependent sensitivity analysis using several indices. There were 317 and 134 patients in cohorts 1 and 2, respectively. Elevated NLR was associated with worse survival (hazard ratio [HR] for death, 1.35 [95% confidence interval 1.19–1.54]; p<0.0001). The optimal NLR cut-off in cohort 1 was dependent on index used and time of assessment: HR values were non-significant at a cut-off of 3.0 (1.34 [0.99–1.32], but significant when the cut-off was 4.0 (1.53 [1.11–2.10]). NLR was linearly related to mortality risk; in subgroup analysis, no significant interaction was found with co-variables or tumor localization overall (cohorts 1+2). Pre-treatment NLR is a useful prognostic tool in patients with metastatic solid tumors, irrespective of primary tumor site, chemotherapy line, age, gender and performance status. However, using an NLR cut-off value for clinical decision-making requires extreme caution.

## Introduction

An inflammatory environment for cancer tumors is thought to promote growth of malignant cells, thus contributing to angiogenesis, mutation, cellular migration and metastasis [1]. Acting via different cytokine pathways, inflammation simultaneously stimulates the production and release of neutrophils from bone marrow, which accumulate and persist in peripheral tissues [2–5] and decreases the production of lymphocytes [6–8]. As a result, increases in the neutrophil-to-lymphocyte ratio (NLR) are indicative of systemic inflammation, which is thought to promote growth of malignant cells, thus contributing to angiogenesis, cellular migration and metastasis [9].

Systemic inflammation is increasingly being recognized as an important determinant of outcome in cancer patients [10–13]. Furthermore, research suggests that markers of systemic inflammation, such as C-reactive protein (CRP), can predict poor prognosis in patients with cancer [13, 14]. There is also a growing body of evidence highlighting a role for NLR as a prognostic indicator in cancer patients [8, 15–26]. However, some of the results of individual studies are inconsistent, particularly across tumor types and disease stage, and there is variety in the NLR cut-off values used to determine increased mortality risk [27]. Nevertheless, NLR is an inexpensive, accessible biomarker that has the potential to be a cost-effective, objective approach to inform clinical decision-making and stratify patients in clinical trials [28].

This study assessed the prognostic value of the pre-treatment NLR in patients with a variety of metastatic solid tumors and clinical characteristics, was based on the Program for Optimization of the Chemotherapy Network (PROCHE) initiative, an innovative prospective oncology-monitoring program designed to improve the quality of patient care [29].

## Materials and methods

This single center, prospective, multi-cohort study was conducted at a teaching hospital in France, with the aim of determining whether an optimal NLR cut-off could be used in the clinic irrespective of tumor type.

### Patient population/Sample

Based on the PROCHE program [29], clinical and biological data were prospectively recorded for patients treated with first-line chemotherapy for metastatic solid tumors in the oncology outpatient department of Georges Pompidou European Hospital between January 2008 and December 2011.

Briefly, all patients with a solid tumor and who are starting chemotherapy in the medical oncology outpatient department could be enrolled in the PROCHE program after having given a signed informed consent. In the PROCHe program, baseline assessment always included neutrophils and lymphocytes count for every patient prior to each cycle and each laboratory analysis were also immediately sent to this dedicated platform.

To be eligible for inclusion in cohort 1 of this study, patients had to have NLR determined in the 28 days before the first cycle of their first line of chemotherapy. To also investigate the prognostic value of NLR in subsequent lines of chemotherapy, patients for whom a NLR was determined in the 28 days before the first cycle of their last line of chemotherapy, and also included in the PROCHE program (cohort 2), were added to the first cohort (overall cohort). All patients provided written informed consent for sharing of their clinical and biological data through the PROCHE program. This specific study was approved by the “Comité de Protection des Personnes (CPP, ie French ethics committee) Ile de France II” on 2 February 2015.

### Statistical analysis

The primary objectives of the study were to assess the influence of NLR on overall survival in patients treated with a first-line chemotherapy for metastatic disease (cohort 1), and to assess the relevance of an optimal NLR cut-off value across multiple tumors. The main secondary objective was to evaluate the dependency of the NLR prognostic value on the number of treatment lines received (cohort 1 + 2).

Overall survival was defined as the time between the first dose of last-line of chemotherapy until patient death or last contact. NLR was collected prior to first dose of last-line of chemotherapy. NLR was used either as a continuous variable or dichotomized using a time-dependent receiver-operated curve [ROC] analysis). Optimality was assessed using several indices: minimal distance to (0.1) point on AUC curve, Youden's J, and concordance probability function, and within each model, bias-corrected bootstrapped confidence intervals of cut-off value were generated from 1000 bootstrap samples. We also investigated the multivariable time-dependency of NLR cut-off using a log-rank test-based method [30]. Non-linear relationship between NLR and survival was evaluated with introduction of penalized cubic splines. Non-proportional hazards between subgroups defined by cut-points were investigated with an additional NLR-time interaction term.

Full models using Cox proportional hazards included the following variables: age, Eastern Cooperative Oncology Group (ECOG) performance status (PS) (0–1 vs. ≥2), and gender (male vs. female). In order to account for the multi-tumor cohort and specification of the model, we plotted the difference in hazard rates for each tumor localization (see online supplement, **S1 Fig**). Given the amplitude of observed differences, instead of a simple final adjustment, it was decided to stratify by tumor localization: breast, lung, urologic, ovarian, head and neck, or other. The influence of the line of chemotherapy on the prognostic value of NLR was investigated in the overall cohort. Statistical analyses were performed using SAS v9.4 (SAS Cary) and R.3.2.3.

## Results

Data for 317 patients with a metastatic solid tumor and an available NLR prior to the first cycle of first-line of chemotherapy (cohort 1) were retrieved from the outpatient database (Table 1). The majority of patients was male, with a good ECOG performance status (PS), and presented mainly with lung or urogenital primary tumors. Lung tumors included only non-small cell lung cancer (NSCLC) treated with a platinum-based chemotherapy and urogenital tumors included mainly castration-resistant prostate cancer (CRPC) treated with docetaxel, and urothelial carcinoma (UC) treated with platinum-based chemotherapy. All patients were naive of chemotherapy for their metastatic disease. Median and mean neutrophil and lymphocyte counts, and their ratio (NLR) collected before the first day of the first cycle of chemotherapy are reported in Table 1.

**Table 1:** Baseline demographics and clinical characteristics.

Patient characteristics	Cohort 1 (n = 317)	Cohort 2 (n = 134)
**Age, years**	64 (58–72)	64 (58–71)
**Male, n (%)**	193 (61)	81 (60)
**Female, n (%)**	124 (39)	53 (40)
**ECOG-PS, n (%):**		
0–1	234 (74	97 (72)
≥2	83 (26)	37 (28)
**Metastatic disease, n (%)**	317 (100)	134 (100)
**Tumour location, n (%):**		
Lung	96 (30)	35 (26)
Urological	81 (25)	48 (36)
Head and neck	51 (16)	10 (8)
Ovarian	40 (13)	14 (10)
Breast	31 (10)	18 (13)
Other	18 (6)	9 (7)
**Previous lines of treatment, n (%)**		
0	317 (100)	0
1	0	61 (46)
2	0	35 (26)
3	0	22 (16)
>3	0	16 (12)
**Neutrophil count, /mm**^**3**^		
Median (Q1-Q3)	4060 (2550–5960)	3687 (2412–5669)
Mean	4709	4460
**Lymphocyte count, /mm**^**3**^		
Median (Q1-Q3)	1421 (987–2001)	1423 (1067–1847)
Mean	1543	1528
**NLR**		
Median (Q1-Q3)	2.68 (1.70–4.93)	2.63 (1.73–4.23)
Mean (standard deviation)	4.21 (4.86)	3.63 (3.34)

Age is reported as median (quartile 1–quartile 3); all other values are number of patients (%) unless otherwise stated.

ECOG-PS, Eastern Cooperative Oncology Group performance status; NLR, neutrophil-to-lymphocyte ratio; Q1, quartile 1; Q3, quartile 3.

In the first cohort of patients to be treated with first line-chemotherapy (n = 317), 186 deaths were observed after a median follow-up of 28 months (95% confidence interval [CI] 25–32), with a median overall survival of 17.6 months (95% CI 14.4–21.0).

The second cohort of patients treated with a later line of chemotherapy (n = 134, cohort 2, Table 1) included a majority of male patients, with good ECOG-PS, and also mainly with lung or urogenital primary tumors. Again, lung tumors included only non-small cell lung cancer (NSCLC) and urogenital tumors included mainly castration-resistant prostate cancer (CRPC) treated with cabazitaxel, and urothelial carcinoma (UC) treated with taxane-based chemotherapy. The majority of patients had received more than one previous line of chemotherapy (range 1 to 6 lines).

After a median follow-up of 28 months (95% confidence interval [CI] 25–32), 84 deaths were observed in this cohort with a median overall survival of 14.0 months (95% CI 10.4–17.0).

### NLR assessed as a continuous variable (cohort 1)

As a continuous variable, increased NLR was significantly associated with worse survival. There was a 35% increase in the risk of death for every standard deviation (SD = 4.86) increase in NLR (hazard ratio [HR] 1.35, 95% CI 1.19–1.54; p<0.0001). ECOG-PS (≥2 vs 0–1) was also significantly associated with worse prognosis (HR 1.54, 95% CI 1.09–2.19; p = 0.01). Analysis with either neutrophil or lymphocyte counts alone, instead of NLR, led to smaller HR values of 1.26 ((95% CI 1.08–1.46); p = 0.002) and 0.80 (95% CI 0.67–0.95; p = 0.011), respectively. No significant time-interaction between continuous NLR and overall survival was seen (p = 0.24). Using a penalized spline to capture non-linear effect of NLR did not improve the prognostic value in cohort 1 (prob(linear effect)<0.0001, prob(non-linear effect) = 0.41).

### NLR and line of chemotherapy (cohort 1 + cohort 2)

Adding cohort 2 to cohort 1 allowed evaluation of the impact of multiple lines of chemotherapy on NLR prognostic value. The median and mean NLR in the overall cohort (n = 451) was 2.65 (Q1-Q3: 1.71–4.78) and 4.04 (SD = 4.47) respectively. Multivariable analysis on the overall cohort including chemotherapy line (first versus later) as an additional co-variable confirmed that high continuous NLR values were significantly associated with worse survival (Table 2). Only ECOG-PS was also significantly associated with worse survival, whereas line of chemotherapy was not (Table 2).There was no interaction between treatment line and continuous NLR (p-value = 0.31).

**Table 2:** Multivariate analysis in the overall cohort of patients receiving chemotherapy (cohort 1 + cohort 2, n = 451) with neutrophil-to-lymphocyte ratio as a continuous variable.

	NLR as a continuous variable *		
Variables	Hazard ratio	95% CI	p-value
**NLR** (1 SD)	1.25	1.12–1.39	< 0.0001
**ECOG-PS** (≥2)	1.51	1.14–2.01	0.004
**Age** (each 10y)	1.06	0.93–1.21	0.39
**Male gender** (Male)	1.07	0.75–1.52	0.72
**Treatment line** (≥2)	1.21	0.93–1.58	0.15

CI, confidence interval; ECOG-PS, Eastern Cooperative Oncology Group performance status; NLR, neutrophil-to-lymphocyte ratio

*SD: standard deviation = 4.47.

### NLR prognostic value by cut-off and length of follow-up

**Fig 1:**
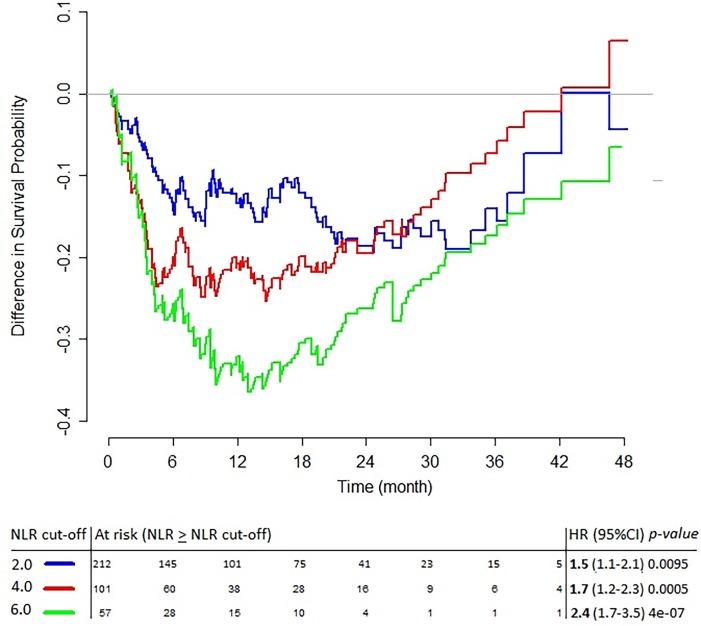
Survival probability for patients with a neutrophil-to-lymphocyte ratio (NLR) at the cut-off value or higher versus below the cut-off value for each unit variation of NLR cut-off (Cox univariate model).

**Fig 2:**
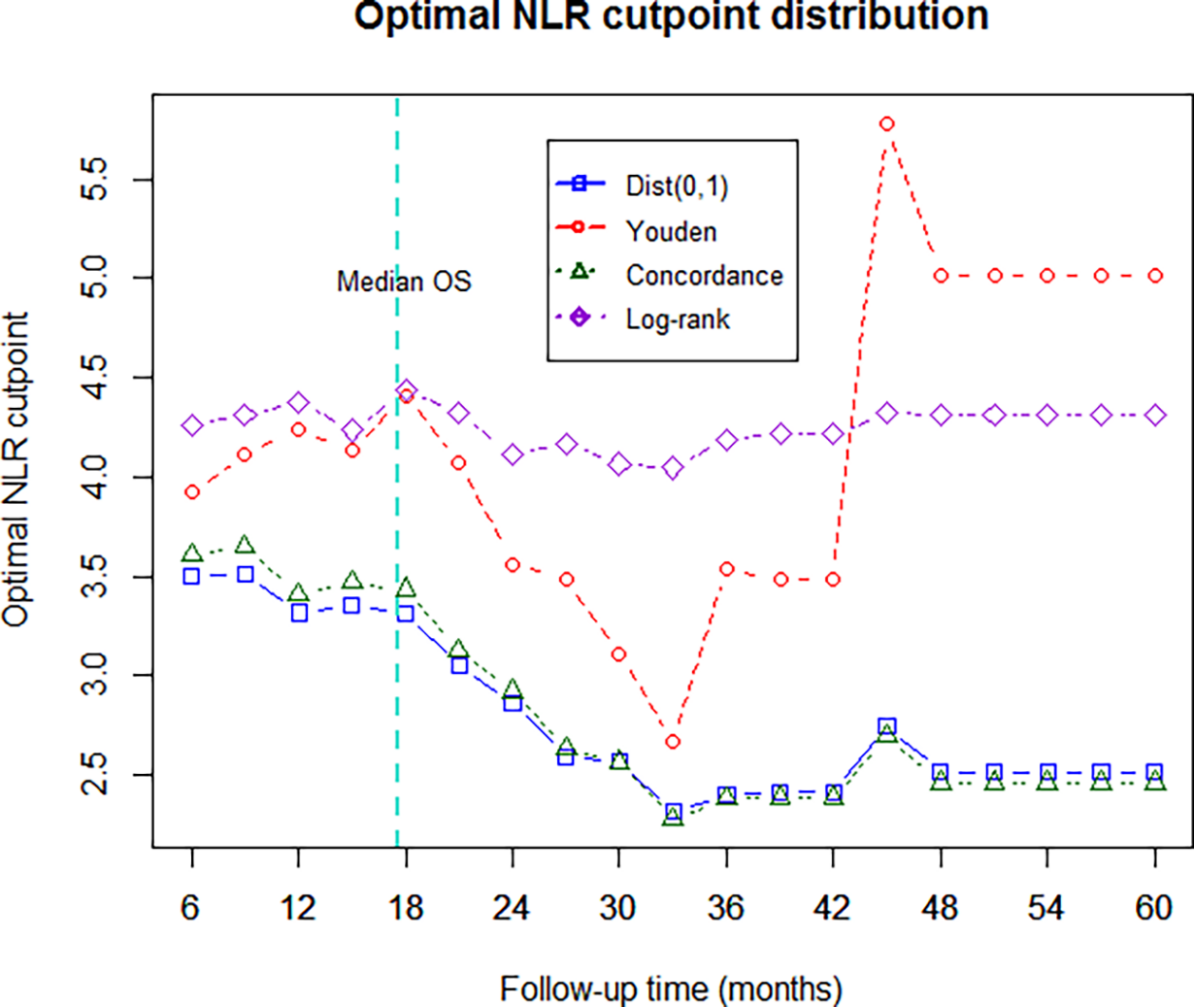
Sensitivity analysis of the neutrophil-to-lymphocyte ratio (NLR) cut-off using 4 different indices. Using the log-rank test-base method, the NLR cut-off value displaying the greatest stability over time was determined to be between 4.0 and 4.5.

In order to assess the clinical usefulness of NLR, i.e. its ability to help the clinician for therapeutic decisions in daily practice, we assessed the “optimality” of NLR cut-off and its prognostic value across multiple tumor types. Hazard ratio values for categorized NLR ranged between 1.5 and 2.4 depending on the cut-off value (Fig 1), higher values being more easily statistically significant due to better discrimination between groups. The NLR effect was dependent on follow-up duration, with largest difference between plus and minus the cut-off observed for patients who survived at least 12 months, and this was true whatever the cut-off value (Fig 1). Adjusted Kaplan-Meier curves for cut-off values of 3.0 and 4.0 illustrate the impact of choosing an arbitrary cut-off (see online supplement, S2A and S2B Fig): despite good separations in both cases, the one-unit increase in the NLR cut-off markedly changed the statistical significance of a score test. Categorizing the NLR also led to a significant time-interaction with overall survival in the Cox model and to a violation of the proportionality of hazard assumption (see online supplement, S3 Fig). Introducing a time-dependent term in the model to correct for this violation increased the NLR effect (see online supplement, S1 Table) suggesting that this assumption should always be investigated and its impact on the NLR effect commented on in studies using an arbitrary cut-off. Sensitivity analysis on the time-dependency of NLR cut-off provided an optimal NLR cut-off between 3.5 and 4.5, with the method based on log-rank testing being the most stable at a NLR cut-off value between 4.0 and 4.5 (Fig 2).

**Fig 3:**
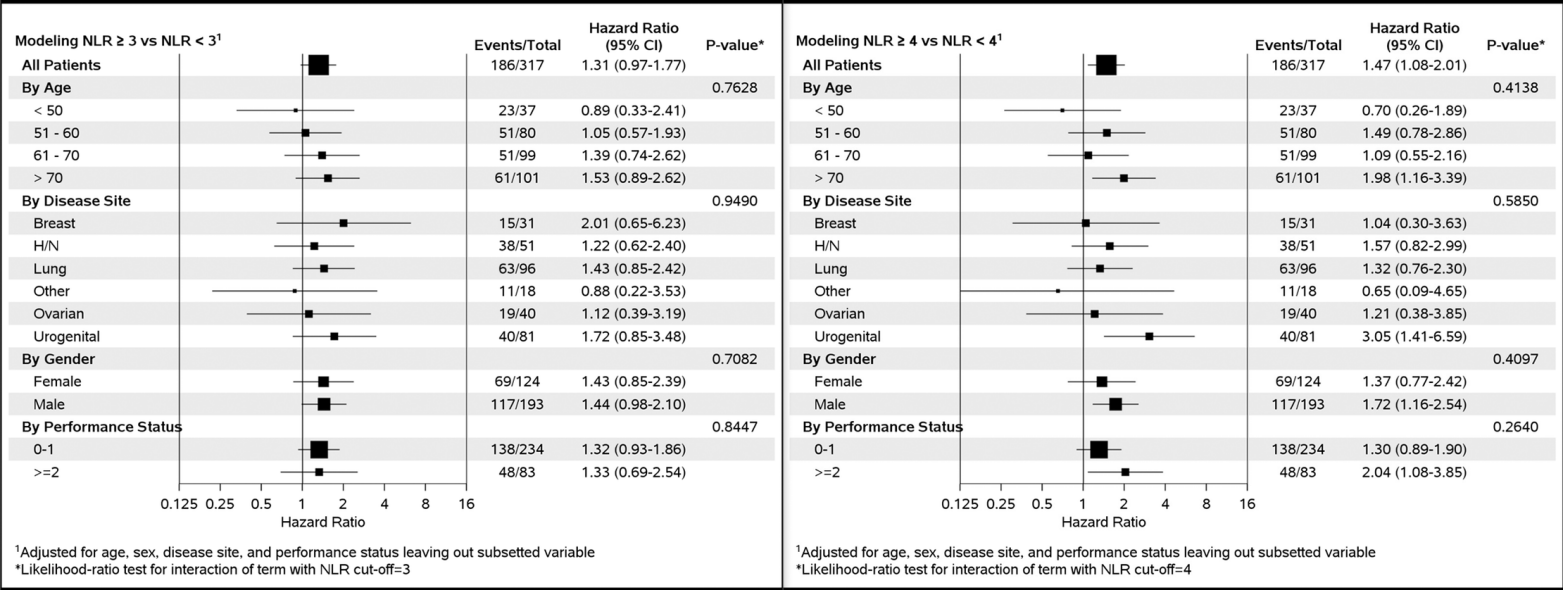
Subgroup analysis illustrating the impact of the choice of neutrophil-to-lymphocyte ratio (NLR) cut-off value; examples with NLR ≥3.0 (**A**) and NLR ≥4.0 (**B**). The model was adjusted for age, sex, disease site and European Co-operative Oncology Group performance status, and the interaction test remained statistically non-significant across the different subgroups, irrespective of the cut-off value, in favour of a persisting negative effect of a high NLR value on prognosis. *Likelihood-ratio txt for interaction of term with NLR cut-off = 3.

The impact of the NLR cut-off was calculated in different patient subgroups (Fig 3). Our point was to illustrate the effect of 1-unit difference in NLR cut-off on OS within each subgroup. In our example comparing a cut-off of 3.0 vs 4.0, the effect of NLR in patients with genitourinary cancer and/or ECOG-PS > = 2 was clinically and statistically significant for 4.0 but not for 3.0. Also considering breast cancer, at cut-off of 3.0, the effect on OS was clinically significant (HR = 2.01) whereas the effect vanished at cut-off of 4.0 (HR = 1.04). Therefore, choosing a cut-off value may also drastically impacts other factors in multivariate analysis. Overall, NLR above the cut-off value was associated with worse survival in most of the subgroups (Fig 3 and Table 3), the effect being more clinically and statistically significant with 4.0 than 3.0, suggesting a comparable prognostic value across several solid tumor types (slightly different values of effect in the forest plot compared with S2A and S2B Fig and S1 Table result from both categorization of age and introduction of tumor localization as adjustment co-variables in the model to get the effects, instead of stratification factors as described in Methods). The heterogeneity test remained statistically non-significant across the different subgroups, which indicates a consistent negative prognostic effect of a high NLR.

**Table 3:** Multivariate analysis in the overall cohort of patients receiving chemotherapy (cohort 1 + cohort 2, n = 451) with neutrophil-to-lymphocyte ratio as dichotomous variable.

Variables	NLR as a dichotomous variable (> cut-off / ≤ cut-off)					
	Cut-off = 3.0			Cut-off = 4.0		
	Hazard ratio	95% CI	p-value	Hazard ratio	95% CI	p-value
**NLR**	1.25	0.97–1.61	0.08	1.42	1.08–1.86	0.01
**ECOG-PS** (≥2)	1.55	1.16–2.06	0.003	1.56	1.18–2.07	0.002
**Age** (each 10y)	1.07	0.93–1.22	0.35	1.07	0.94–1.22	0.32
**Male gender** (Male)	1.05	0.73–1.51	0.78	1.04	0.72–1.48	0.85
**Treatment line** (≥2)	1.24	0.95–1.61	0.11	1.23	0.94–1.60	0.12

CI, confidence interval; ECOG-PS, Eastern Cooperative Oncology Group performance status; NLR, neutrophil-to-lymphocyte ratio

## Discussion

To the best of our knowledge, this is the first study with individual patient data to show that elevated NLR is associated with worse survival in patients with various metastatic solid tumors, irrespective of the primary tumor site, line of chemotherapy, age, gender and ECOG-PS. In order to account for the diversity of tumor types, stratification allowed NLR effect to be calculated first within each stratum before getting an overall result.

Current literature show that interest in NLR over the last decade has grown rapidly. An elevated NLR has been shown to be associated with reduced overall survival in patients with a variety of different individual tumor types [8, 15–26], disease stages (localized or metastatic) and treatments (chemotherapy or radiotherapy) [27]. However, very few previous trials have included patients with different tumor types [31, 32] as we did in this study. Inclusion of patients with a variety of tumor types allows us to investigate any differential prognostic value of NLR. We found that elevated NLR was associated with poor prognosis independently of tumor type. Thus, our results confirm data from a large meta-analysis suggesting that elevated NLR was a strong predictor of poor survival across all tumor types [28]. Because the meta-analysis was based on published data, the influence of other prognosis parameters such as ECOG-PS or age on the prognostic value of NLR was not reported. In addition, the influence of the line of chemotherapy was not assessed. In our study, we show that NLR prognostic value was not influenced by ECOG-PS, age, gender or tumor location. In addition, in another cohort of 134 patients treated with later lines of chemotherapy, we show that NLR prognostic value was still independently associated with worse survival. These results are in line with the fact that the prognostic value of NLR has been demonstrated in a wide range of disease stages, from localized disease to patients receiving palliative care [33] or terminally ill [34].

Furthermore, in contrast to the majority of previous studies, we report the prognostic value of NLR as a continuous variable to avoid the loss of information inherent in data-driven categorization. We found that an increase in NLR of 1 standard deviation (SD) increased the risk of death by 35% (p<0.0001) after adjustment for other prognostic factors (ECOG-PS, age, gender, tumor type); we believe that “SD increase” may be a better indicator for decision purposes than binary cut-offs. In addition, we also compared the prognostic value of NLR with that of neutrophil or lymphocyte count alone. We found that an elevated neutrophil count significantly increased the risk of death by 26% whereas an elevated lymphocyte count decreased the risk of death by 20%, the magnitude of impact being lower than for NLR (35%).

Published data suggest high variability in the optimal NLR cut-off value, ranging between 1.9 and 9.21 [28, 34]. In order to identify the optimal NLR cut-off value in our study, we performed extensive sensitivity analyses. We found that in patients with a variety of solid tumors, the “optimal” pre-treatment cut-off was between 3.5 and 4.5. Patients with an NLR ≥4.0 had a 53% increased risk of death (p<0.008, Fig 3B) compared with those who had an NLR value <4.0, after adjustment for age, gender, disease site, and Eastern Co-operative Oncology Group performance status. In the largest meta-analysis, median cut-off for high NLR was 4.0 (range 1.9–7.2) and patients with NLR above this cut-off had an 81% increased risk of death (p<0.001) [28], a value that is similar to our findings. Nevertheless, the optimal NLR cut-off was highly variable based on the time at which survival was assessed and the indices used for optimality assessment, questioning the real-life practical “decisional value” of such a cut-off.

Our study is probably the first to show that categorizing the NLR resulted in a significant time-interaction and that introducing a time-dependent term in the model could modify the NLR effect. Indeed, NLR effect was even more pronounced after introducing time-dependent term suggesting that this should always be included in future investigations and its impact on the prognostic value of NLR discussed in studies using an arbitrary cut-off. Moreover we report again for the first time that the index used for optimality assessment of the time-dependent NLR cut-off had an impact on the resulting optimal value. We found that the log-rank method was the most stable across time and gave an optimal value between 4.0 and 4.5. Given our results on the variability of NLR effect, we believe that no standardized cut-off values may be reliable enough to be used in the clinics for decision purposes.

Mechanisms underlying the prognostic value of NLR remain unclear. It is widely considered that NLR reflects a balance between inflammatory status, represented by neutrophils, and adaptive immunity, represented by lymphocytes [9]. Thus, an increase in NLR could reflect an increase in inflammation status or a decrease in anti-tumor immunity, or both. Neutrophils have been shown to produce pro-inflammatory cytokines such as interleukin (IL)-6, which is a major mediator of the hepatic acute-phase response and induces production of acute-phase proteins including CRP, a well-known circulating marker of inflammation. High CRP has also been reported to predict poor prognosis in many tumor types [14]. Consistent with these observations, inflammation is a well-known indicator of tumor progression [9].

A high density of tumor-infiltrating lymphocytes (TIL) has been shown to be strongly associated with a good prognosis in the majority of solid tumors [35]. Nevertheless, no correlation has been documented between density of TIL and circulating lymphocyte count. In fact, the example of renal cell carcinoma where a high density of CD8+ TIL [35] and a low circulating lymphocyte count [36] are associated with a poor prognostic shows the opposite. Few studies have specifically analyzed circulating leucocytes in patients with cancer. Ohki et al. used flow cytometry to characterize peripheral blood mononuclear cells (PBMC) from patients with various tumor types compared with healthy donors [37]. They showed a significant increase in the percentage of myeloid-derived suppressor cells (MDSCs) in PBMC from patients versus healthy donors. In addition, they reported a strong correlation between circulating MDSC and NLR.

Important strengths of our study are its prospective collection of clinical and biological data thanks to the PROCHE program, which had already demonstrated its usefulness [29]. Another strength is the inclusion of patients with a wide range of different solid tumors, which increases the clinical applicability of our results.

Our study has some limitations that should be noted. Firstly, this is a single center study. Nevertheless this point strengthens our results because the management of all patients was highly homogenous and we are sure that data collected through the PROCHE program are reliable. Secondly, we did not look at comorbidities and concomitant medications meaning that any influence of these factors on hematologic variables could not be taken into account. Nevertheless, we verified that no patient had received any growth factor treatment and/or any corticosteroid at the time of NLR collection, which could have an obvious influence on the NLR value. Thirdly, NLR was not assessed during cancer treatment; therefore, data on changes in this biomarker over time and their predictive value for response to therapy could not be determined. Some studies have suggested that the early normalization of the NLR during chemotherapy was a surrogate marker for better survival [38–40].

In conclusion, the results of this study add to knowledge about NLR as an indicator of mortality risk in patients with cancer, providing better understanding of the consistency and magnitude of the prognostic information given by this biomarker. There is strong evidence that the increased mortality risk associated with high NLR is independent of the primary tumor site, line of chemotherapy, age, gender and ECOG-PS. Therefore, NLR alone might be used for prognosis across a wide range of solid tumor types in clinical practice. Nevertheless, challenges discussed above regarding the use of any arbitrary cut-off restrict its use. To overcome this limit and to move forward we are convinced that we have to monitor the variation of the NLR during systemic therapy in order to define patients who will not respond.

## Supporting information

S1 Table Multivariate analysis of effect of continuous and categorised neutrophil-to-lymphocyte ratio values (cut-off 3.0 and 4.0) in patients receiving first-line chemotherapy (cohort 1, n = 317). (DOCX)

S1 Fig Hazard rate for death by tumor type. H&N, head and neck.
(DOCX)

S2 Fig Adjusted Kaplan-Meier curves for the relationship between the neutrophil-to-lymphocyte ratio (NLR) as a categorical variable and overall survival (OS) in cohort 1 (n = 317) for NLR cut-off values of 3 (**A**) and 4 (**B**). Adjustment was made for age, gender, disease site, and Eastern Co-operative Oncology Group performance status. CI, confidence interval; HR, hazard ratio.
(DOCX)

S3 Fig Time-varying value of the neutrophil-to-lymphocyte ratio (NLR) effect in the Cox model. (DOCX)

S1 Database Study full database. (XLSX)
