# Supplementary material for: Optimal cut-off for neutrophil-to-lymphocyte ratio: Fact or Fantasy? A prospective cohort study in metastatic cancer patients
Source: PLoS One. 2018 Apr 6;13(4):e0195042. doi: 10.1371/journal.pone.0195042 (PMC5889159; doi:10.1371/journal.pone.0195042)
Supplement: S1 Table — (DOCX) [file pone.0195042.s001.docx]

**Table S1.** Multivariate analysis of effect of continuous and categorised neutrophil-to-lymphocyte ratio values (cut-off 3.0 and 4.0) in patients receiving first-line chemotherapy (cohort 1, n=317)

| **Variables** | **Hazard ratio** | **95% CI** | **p-value** | **Hazard ratio** | **95% CI** | **p-value** |
| --- | --- | --- | --- | --- | --- | --- |
|  | **NLR as a continuous variable**  (per 1 SD* increase) | | | **NLR as dichotomous variable**  NLR cut-off: 3.0, 4.0  HR (> cut-off / ≤ cut-off) | | |
| **NLR** | 1.35 | 1.19-1. 54 | <0.0001 | 3.0: 1.34 | 0.99-1.82 | 0.058 |
|  |  |  |  | 4.0: 1.53 | 1.11-2.09 | 0.008 |
| **NLR**  **+ time-interaction** | 1.42 | 1.21-1.66 | 0.34 (NS) | 3.0: 2.36 | 1.39-3.99 | 0.0006 |
|  |  |  |  | 4.0: 3.24 | 1.86-5.65 | 0.002 |

CI, confidence interval; ECOG-PS, Eastern Cooperative Oncology Group performance status; NLR, neutrophil-to-lymphocyte ratio; SD, standard deviation.

*SD = 4.86
