# Supplementary figures and images for: Optimal cut-off for neutrophil-to-lymphocyte ratio: Fact or Fantasy? A prospective cohort study in metastatic cancer patients
Source: PLoS One. 2018 Apr 6;13(4):e0195042. doi: 10.1371/journal.pone.0195042 (PMC5889159; doi:10.1371/journal.pone.0195042)

**S1 Fig.**


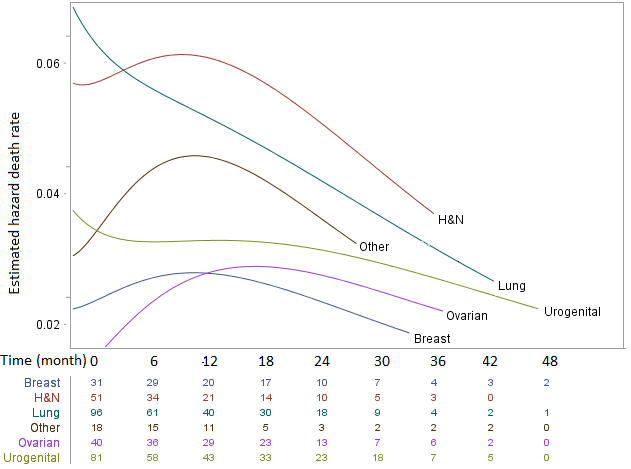

Supplement: S1 Fig — H&N, head and neck. (DOCX) [file pone.0195042.s002.docx]

**S2 Fig.** **A and B**

**A.**


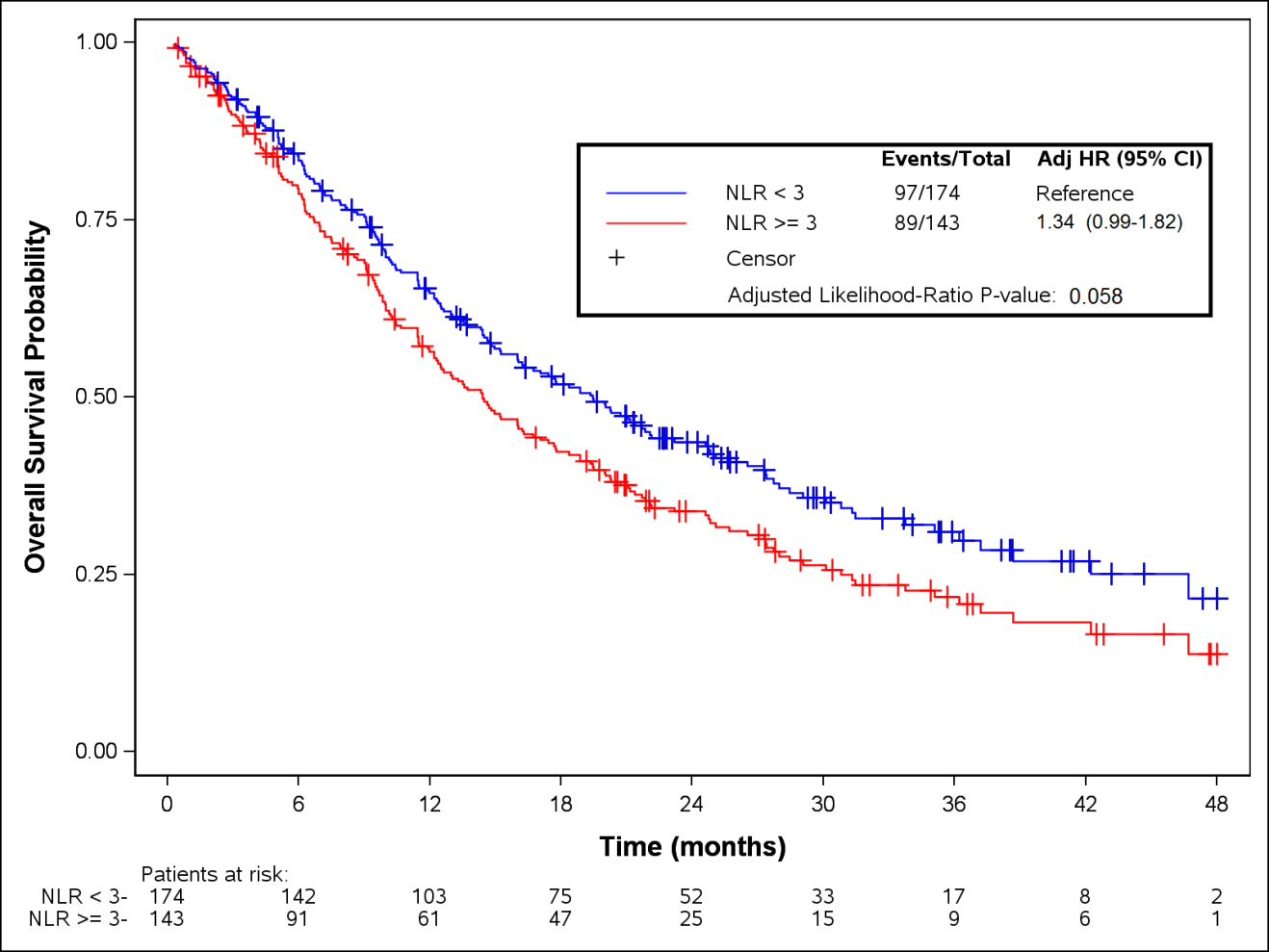


**B.**


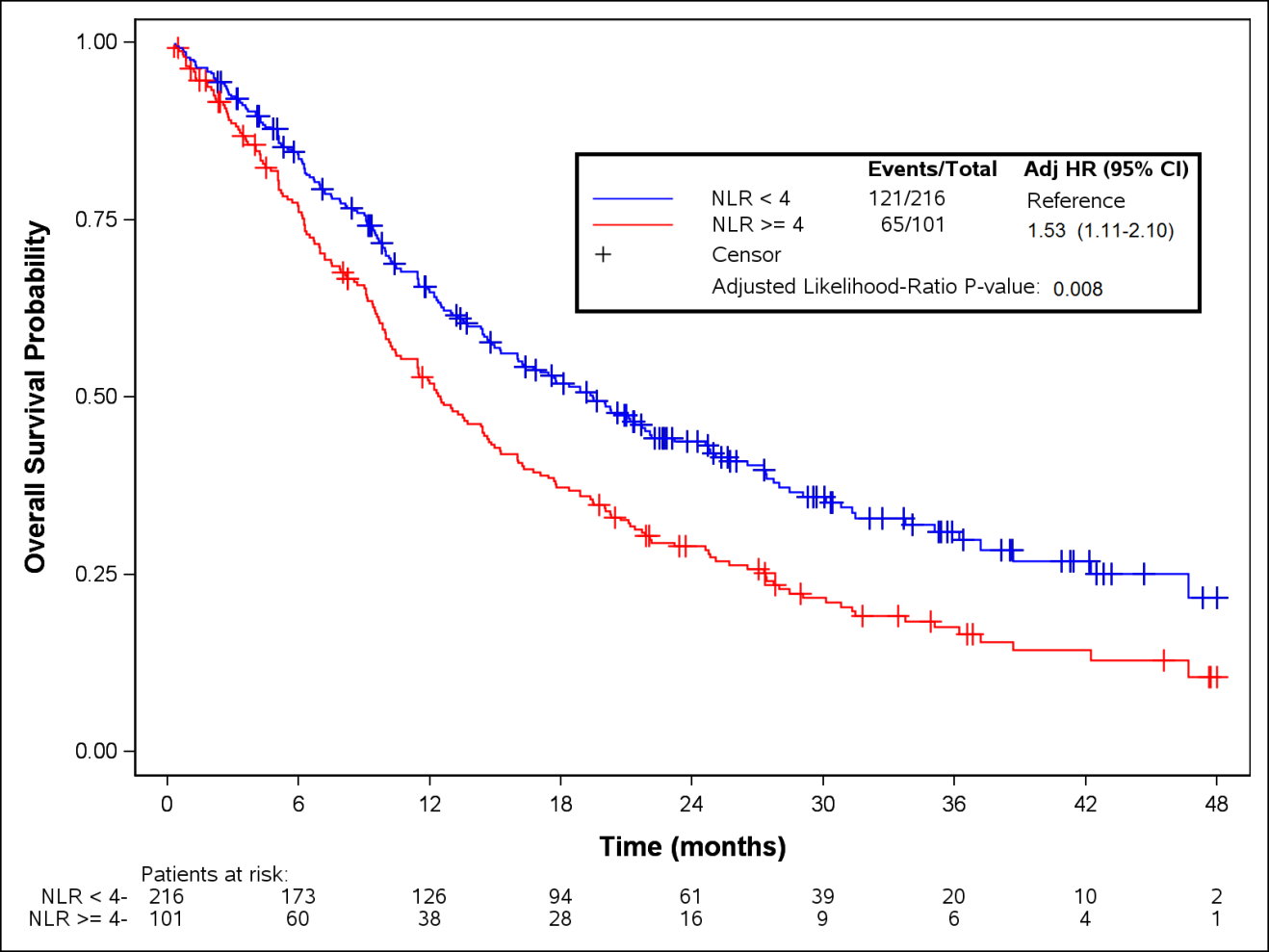

Supplement: S2 Fig — Adjusted Kaplan-Meier curves for the relationship between the neutrophil-to-lymphocyte ratio (NLR) as a categorical variable and overall survival (OS) in cohort 1 (n = 317) for NLR cut-off values of 3 (A) and 4 (B). Adjustment was made for age, gender, disease site, and Eastern Co-operative Oncology Group performance status. CI, confidence interval; HR, hazard ratio. (DOCX) [file pone.0195042.s003.docx]

**S3 Fig.**


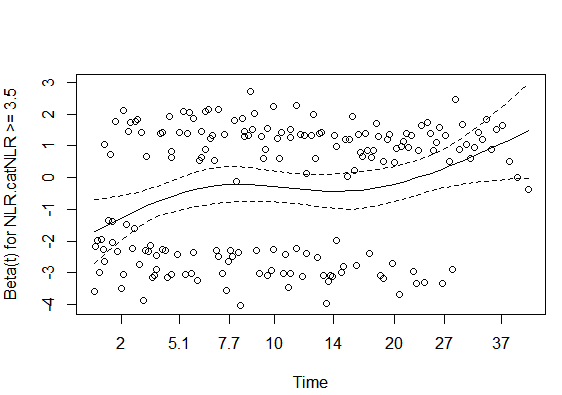

Supplement: S3 Fig — (DOCX) [file pone.0195042.s004.docx]
